# Seasonal Patterns of Mixed Species Groups in Large East African Mammals

**DOI:** 10.1371/journal.pone.0113446

**Published:** 2014-12-03

**Authors:** Christian Kiffner, John Kioko, Cecilia Leweri, Stefan Krause

**Affiliations:** 1 Centre For Wildlife Management Studies, The School For Field Studies, Karatu, Tanzania; 2 Tanzania Wildlife Research Institute, Arusha, Tanzania; 3 Lübeck University of Applied Sciences, Lübeck, Germany; Universidad Carlos III de Madrid, Spain

## Abstract

Mixed mammal species groups are common in East African savannah ecosystems. Yet, it is largely unknown if co-occurrences of large mammals result from random processes or social preferences and if interspecific associations are consistent across ecosystems and seasons. Because species may exchange important information and services, understanding patterns and drivers of heterospecific interactions is crucial for advancing animal and community ecology. We recorded 5403 single and multi-species clusters in the Serengeti-Ngorongoro and Tarangire-Manyara ecosystems during dry and wet seasons and used social network analyses to detect patterns of species associations. We found statistically significant associations between multiple species and association patterns differed spatially and seasonally. Consistently, wildebeest and zebras preferred being associated with other species, whereas carnivores, African elephants, Maasai giraffes and Kirk's dik-diks avoided being in mixed groups. During the dry season, we found that the betweenness (a measure of importance in the flow of information or disease) of species did not differ from a random expectation based on species abundance. In contrast, in the wet season, we found that these patterns were not simply explained by variations in abundances, suggesting that heterospecific associations were actively formed. These seasonal differences in observed patterns suggest that interspecific associations may be driven by resource overlap when resources are limited and by resource partitioning or anti-predator advantages when resources are abundant. We discuss potential mechanisms that could drive seasonal variation in the cost-benefit tradeoffs that underpin the formation of mixed-species groups.

## Introduction

Group living in animals has attracted extensive attention in behavioural, ecological and evolutionary studies and is thought to have evolved from trade-offs between fitness relevant costs and benefits [1]–[6]. Typical benefits associated with living in a group include reduced predation risk through several mechanisms [7]–[11]. Living in a group may also affect the individual foraging success [2], [3], [5] and may either enhance or decrease the transmission rate of disease agents or parasites between individuals [12], [13].

A special case of group living occurs if individuals of different species form a group. Such mixed species groups have long been recognized in a variety of animal communities: mixed species groups have been described in fish communities [14]–[16], avian assemblages [17]–[19] and in marine and terrestrial mammals [20]. Among mammals, mixed species groups have been extensively described in primate communities [21]–[24] and in cetaceans [25]–[27]. Mixed species groups have also been reported in other terrestrial mammals, for example in bovid species in Africa and Asia [9], [28], [29]. With few examples, most studies on mixed species groups have so far focussed on selected species within a community [30] and there are few published examples of cross-taxon mixed groups [31]–[34]. Generally, mixed species groups are associated with similar costs and benefits as single species groups. However, there are at least three major differences between these group types [30]. First, different species might exploit resources in different ways and hence heterospecific group members may impose less competition for food than conspecific group members. The highly differentiated feeding partitioning of grass height observed in grazers inhabiting East African savannah ecosystems is a prominent example for such niche portioning that facilitates co-existence of several grazing species [35]–[37]. Second, species vary in their ability to acquire information in the environment and therefore individuals associated with other species may be more effective in detecting a range of threats or resources [38]. Third, pathogen transmission is mediated by species composition; some pathogens may spill over from different hosts while other hosts may dilute the pathogen infection risk [39]. As a result of these three aspects, group size trade-offs may be altered in mixed-species groups possibly leading to group size differences in single- versus mixed-species groups.

A common approach to describe and analyse social structure in animal communities are social network analyses which allow investigating relationships between single entities (individuals or species) in the context of the overall population or community structure [40]. Recent approaches attempt to identify factors and mechanisms that underpin or explain the observed social structure [41], [42].

East African savannah ecosystems boast a high diversity and density of large herbivores and carnivores [43], [44]. Yet, the abundance and species richness of wildlife has declined severely in the last four decades [45]–[48]. In order to establish a benchmark of mixed species groups in these mammal communities, we embarked on an extensive study and describe species networks in the Serengeti-Ngorongoro and the Tarangire-Manyara ecosystems. Relatively high mammal species diversity and density and good visibility offered a unique setting for identifying mixed species groups of the most common large mammal species with modern network-analysis tools [40], [49], [50]. Beyond investigating (1) whether mixed species groups follow non-random patterns, (2) if grouping patterns were consistent across seasons and ecosystems, and (3) if group sizes depended on association patterns, we analysed (4) if relative abundance of species or preferences of species affect the structure of animal networks.

## Methods

### Study areas

**Figure 1:**
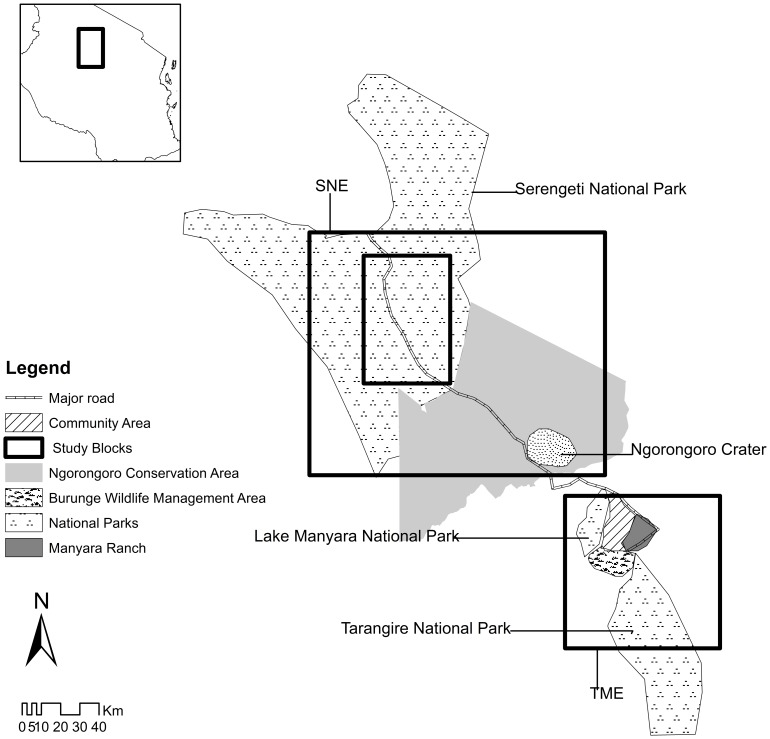
Map of the study area. Sampling was conducted in the Serengeti-Ngorongoro Ecosystem (SNE) and the Tarangire-Manyara Ecosystem (TME) in Northern Tanzania; the inlet in the top left depicts the approximate location of the study area in Tanzania.

Field work was conducted in two savannah ecosystems in Northern Tanzania; the Tarangire-Manyara and the Serengeti-Ngorongoro ecosystem (from here on TME and SNE, respectively) (Fig. 1). TME covers approximately 20,000 km^2^ and is characterized by a patchwork of protected areas, interspersed with settlements, intensively farmed areas and extensively used pastoral areas [51]. Within TME, mammal groups were assessed in two fully protected areas where no consumptive natural resource utilisation is allowed: the northern sector of Tarangire National Park (centred at: S 3°46′06.20, E 36.01′51.20), and Lake Manyara National Park (S 3°30′50.70, E 35°48′36.54). Mammal groups were also assessed in the following multiple-use areas: Manyara Ranch Conservancy (S 3°33′45.22, E 35°58′44.32), Mto Wa Mbu Game Controlled Area (S 3°24′28.40, E 35°55′24.99) and Burunge Wildlife Management area (S 3°42′23.00, E 35°52′20.75). In SNE, mammal groups were assessed in the Ngorongoro Conservation Area and in Serengeti National Park. Sampling in SNE was conducted in the Ngorongoro crater (S 3°10′40.20, E 35°34′47.49) and along the main road towards Nabi gate (S 3°01′19.15, E 35°19′01.72). Within the Serengeti national park, species groups were assessed in the southern and south-western short grass areas and in the central woodland areas (S 2°27′28.22, E 34°49′55.79) (Fig.1).

Both ecosystems are characterized by spatially and temporally variable rainfall patterns. In TME rainfall ranges from 430–850 mm [52], [53]; precipitation mainly falls in the wet season (November-May). In SNE, the annual precipitation ranges from 500 mm in the southeast to 1200 mm in the northwest [54]. In both ecosystems, many species respond to variable rainfall and food resource availability with large-scale migrations or by small scale adjustments of their distribution; for TME see [55], and for SNE see [56]. To account for these seasonal differences, we assessed mammal groups separately during the rainy season (February-April) and during the dry season (June-August) 2012.

### Ethics statement

Research permission to conduct this observational study in all study areas was granted by Tanzanian Wildlife Research Permit No. 012-241-NA-2012-57.

### Mammal group assessment

Mammal groups were assessed along roads and tracks from 4WD vehicles. We observed animals on 30 (21) observer days in TME during wet (dry) season, and 39 (38) in SNE during wet (dry) season in 2012. Two to eight observers trained in species identification scanned for wild mammals up to a range of 500 m from the vehicle. When wildlife was encountered, the vehicle was stopped, animals were identified to species level and the surrounding was scanned for other species; at far distances binoculars were used. Species assessed in this study included 26 commonly encountered mammalian species in five orders (Table 1): *Artiodactyla* (15 species), *Carnivora* (6 species, the three sympatric jackals were combined), *Perissodactyla* (1 species), *Primates* (3 species), and *Proboscidae* (1 species). We defined conspecific and heterospecific individuals within 50 m of each other to be in a group [57], a common cut off distance for defining mixed species groups [20]. Distances were estimated by eye and observers frequently used a laser rangefinder to calibrate their distance estimates. All independent sightings of single and mixed species groups, called “clusters” in the following, were recorded consistently and were used to create species occurrence matrices for each ecosystem and season. In these 4 matrices, a “1” in row “*i*” of column “*j*” denotes that species “*j*” was present in cluster “*i*”, while a “0” marks its absence. For each sighting we also recorded the habitat type (grassland, open bushland, closed bushland, woodland, riverine vegetation, shrubland). We recorded a total of 5403 clusters: 858 (438) in TME during the wet (dry) season, and 2324 (1783) in SNE during the wet (dry) season.

**Table 1:** Summary of species considered for analyses listed by taxonomic order.

Order	Common name	Abbreviation	Scientific name	Feeding guild	ESW (m)
***Artiodactyla***	**Hippopotamus**	**Hi**	***Hippopotamus amphibious***	**Grazer**	**275**
***Artiodactyla***	**Maasai giraffe**	**Gi**	***Giraffa camelopardalis***	**Browser**	**255**
***Artiodactyla***	**Cape buffalo**	**Bu**	***Syncerus caffer***	**Grazer**	**405**
***Artiodactyla***	**Eland**	**El**	***Taurotragus oryx***	**Mixed**	**457**
*Artiodactyla*	Waterbuck		*Kobus ellipsiprymnus ellipsiprymnus* (TME)/*defassa* (SNE)	Grazer	154
***Artiodactyla***	**Wildebeest**	**Wb**	***Connochaetes taurinus***	**Grazer**	**405**
***Artiodactyla***	**Coke's hartebeest**	**Ha**	***Alcelaphus buselaphus***	**Grazer**	**275**
***Artiodactyla***	**Topi**	**To**	***Damaliscus korrigum***	**Grazer**	**275**
*Artiodactyla*	Warthog		*Phacochoerus africanus*	Omnivore	135
*Artiodactyla*	Impala		*Aepyceros melampus*	Mixed	100
***Artiodactyla***	**Grant's gazelle**	**GG**	***Nanger granti***	**Mixed**	**230**
*Artiodactyla*	Bohor reedbuck		*Redunca redunca*	Grazer	100
*Artiodactyla*	Bushbuck		*Tragelaphus scriptus*	Browser	31
***Artiodactyla***	**Thomson's gazelle**	**TG**	***Eudorcas thomsonii***	**Mixed**	**340**
*Artiodactyla*	Kirk's dik dik		*Madoqua kirkii*	Browser	27
*Carnivora*	African lion		*Panthera leo*	Carnivore	50
*Carnivora*	Spotted hyena		*Crocuta crocuta*	Carnivore	50
*Carnivora*	Cheetah		*Acinonyx jubatus*	Carnivore	50
*Carnivora*	Leopard		*Panthera pardus*	Carnivore	50
*Carnivora*	Serval		*Leptailurus serval*	Carnivore	25
*Carnivora*	Jackals		*Canis adustus, C. aureus & C. mesomelas*	Omnivore	25
***Perissodactyla***	**Plains zebra**	**Ze**	***Equus quagga***	**Grazer**	**275**
*Primates*	Olive baboon		*Papio anubis*	Omnivore	83
*Primates*	Vervet monkey		*Chlorocebus pygerythrus*	Omnivore	21
*Primates*	Blue monkey		*Cercopithecus mitis*	Omnivore	18
*Proboscidae*	African elephant		*Loxodonta africana*	Mixed	71

ESW is the effective strip width, i.e. the distance from the transect for which as many animals were detected beyond that distance as were detected within that distance [62]. Species with high detectability used for network construction are highlighted in bold.

### Overview of methods used for the analyses

Different methods are necessary for the investigation of the four questions listed in the introduction. For (1) and (2), we randomised the observed group compositions and used as test statistics the sum of squares of co-occurrences to test for species-specific associations, the numbers of co-occurrences to test for specific association dyads, and the node strength to test if some species were typically more heterospecifically-gregarious than expected based on our observation data. For (3), we permuted the observed group sizes to determine if heterospecific associations were traded-off against conspecific associations and if group sizes depended on the presence of preferred other species. For (4), we measured node betweenness (a measure of importance in the flow of information or disease [40]) to determine which species were relatively more important in these mixed-species communities, and used a simulation routine to infer the importance of abundance or choice in determining these patterns. The following sections explain these methods in more detail.

### Testing for species-specific association patterns

These tests follow Besag and Clifford ([58]; see [49] for a description of the correct implementation), hereafter referred to as BC test, and randomize the occurrence matrix while keeping constant the number of occurrences of each species, and the number of clusters and their sizes (i.e. the numbers of different species in the clusters). This is crucial because detection probabilities were species-specific (Table 1) and in order to include all 26 species we needed to use a test that takes the observed abundances into account rather than making any assumptions about the unknown real abundances. To test for associations among species we chose as our test statistic the sum of squares of the numbers of co-occurrences of each pair of species [49]. This test statistic yields large values, if there is a general tendency for species to co-occur with specific other species. Large values of the sum of squares may be caused by only a subset of the pairs of species. Therefore, we also tested for specific association dyads where we chose the number of co-occurrences of the respective pair of species as our test statistic. Here, large values of the test statistic mean that the respective pair of species co-occurs more often than expected given the context of all observed clusters. The usage of absolute numbers in our test statistics is justified by the randomization procedure which keeps the number of occurrences of each species constant. This means, for example, that for rarely occurring species a small number of co-occurrences may yield significant associations, while for frequently occurring species a high number of co-occurrences may not necessarily indicate significant relationships.

To find out whether species show specific tendencies regarding their general co-occurrence with other species we used the node strength as test statistic. The computation of the node strength requires the construction of a network as a first step. We defined the species as nodes of this network and added an edge with weight *w_i,j_* between the nodes *i* and *j*, if species *i* and *j* co-occurred *w_i,j_*>0 times in the observed clusters. After each randomization of the occurrence matrix a network was constructed. This approach generates network samples that are equally likely in the absence of associations, given the observed numbers of clusters, the cluster sizes, and the numbers of occurrences per species. The reason for using weighted networks is that in our study any edge is possible and the relevant information is contained in the weights rather than in the existence of an edge. The node strength *s_i_* of node *i* measures the total weight of all edges connected to *i*. More formally, *s_i_* =  Σ_j
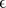
*Ci*_ *w_i,j_*, where *C_i_* is the set of all nodes (i.e. species) that are connected with *i* via some edge. In our case, the node strength measures the tendency of a species to occur in mixed species groups.

### Testing for group size patterns

To determine if heterospecific associations were traded-off against conspecific associations we tested whether the group sizes of a species generally differed between occurrences in multi- and in single-species clusters. Additionally, we tested whether group sizes of a given species depended on the presence of a significant dyadic association with a specific other species. The tests were performed by permuting the group sizes of the observed occurrences of each species separately and using the median group size as test statistic. More precisely, if species *s* was observed *k_s_* times and *g_1_*(*s*),…, *g_ks_*(*s*) are the group sizes of each occurrence of species *s*, then a randomisation step consisted of permuting these lists for each species. This procedure takes the observed abundance of each species into account by keeping constant the sum of the group sizes per species.

### Analysing the betweenness in a multi-species network

The above described tests analyse patterns caused by preferences among the species. Significance regarding the number of co-occurrences of two species provides strong evidence for a non-random association between them caused by the species' behaviour. However, this only means that they co-occur more often than expected under the assumption of “no association” and not necessarily that the absolute number of their co-occurrences is high. It can also be important to look at structural patterns regardless of whether they were caused by preferences or just by random encounters. If a pathogen spreads quickly among individuals of a certain species, individuals of other species might get infected just because both species occur in large numbers and therefore encounter each other frequently regardless of specific preferences. A common tool for this kind of analysis is networks [59], [60]. For our purposes we constructed networks as described previously and analysed the node betweenness of the species. The node betweenness of a node *i* measures the extent to which shortest paths between other nodes run through *i*. It is a standard measure of centrality and is often used as an indicator of a node's relevance regarding exchange between other nodes [40], [61]. However, when trying to draw absolute conclusions from networks, e.g. that some species have a high node betweenness, two issues arise. Firstly, networks should not contain artefacts caused by our observation method. In particular, the number of sightings must not be (strongly) affected by variable detectabilities. Therefore, we retained only 10 species (9 species in TME) with high detectability (Table 1). Species-specific detection probabilities were estimated using a separate distance sampling survey conducted in TME (unpublished data and supplemented with [62]). We considered species as highly detectable if their effective strip width [63]) exceeded 230 m (Table 1). This data reduction was performed using an independent criterion and is thus in contrast to problematic thresholding procedures that remove weak links or rarely observed nodes [42]. Secondly, the amount of data needs to be sufficient to construct robust networks [64]. Adding or removing edges can have strong effects on measures like the node betweenness. It is therefore necessary to investigate to what degree potential variants of the sampled network affect relevant network metrics. In our case, it does not seem appropriate to directly modify a network because it is almost impossible to judge how likely it is for an edge to have a certain weight or to be present at all, nor is it useful to randomize the observed networks because randomizations only use the observed data and therefore do not allow conclusions about the networks' robustness. Since we did not observe networks but constructed them based on observations of clusters, i.e. took observational snapshots of a process, we used a simulation described in the following section. We repeatedly generated sets of clusters that followed basic patterns derived from our observations, such that each set represented a potential observation. Based on each simulated set of clusters we constructed a network and computed the node betweenness. This allowed us to judge the robustness of this measure in our observed networks.

### Network simulations

**Figure 2:**
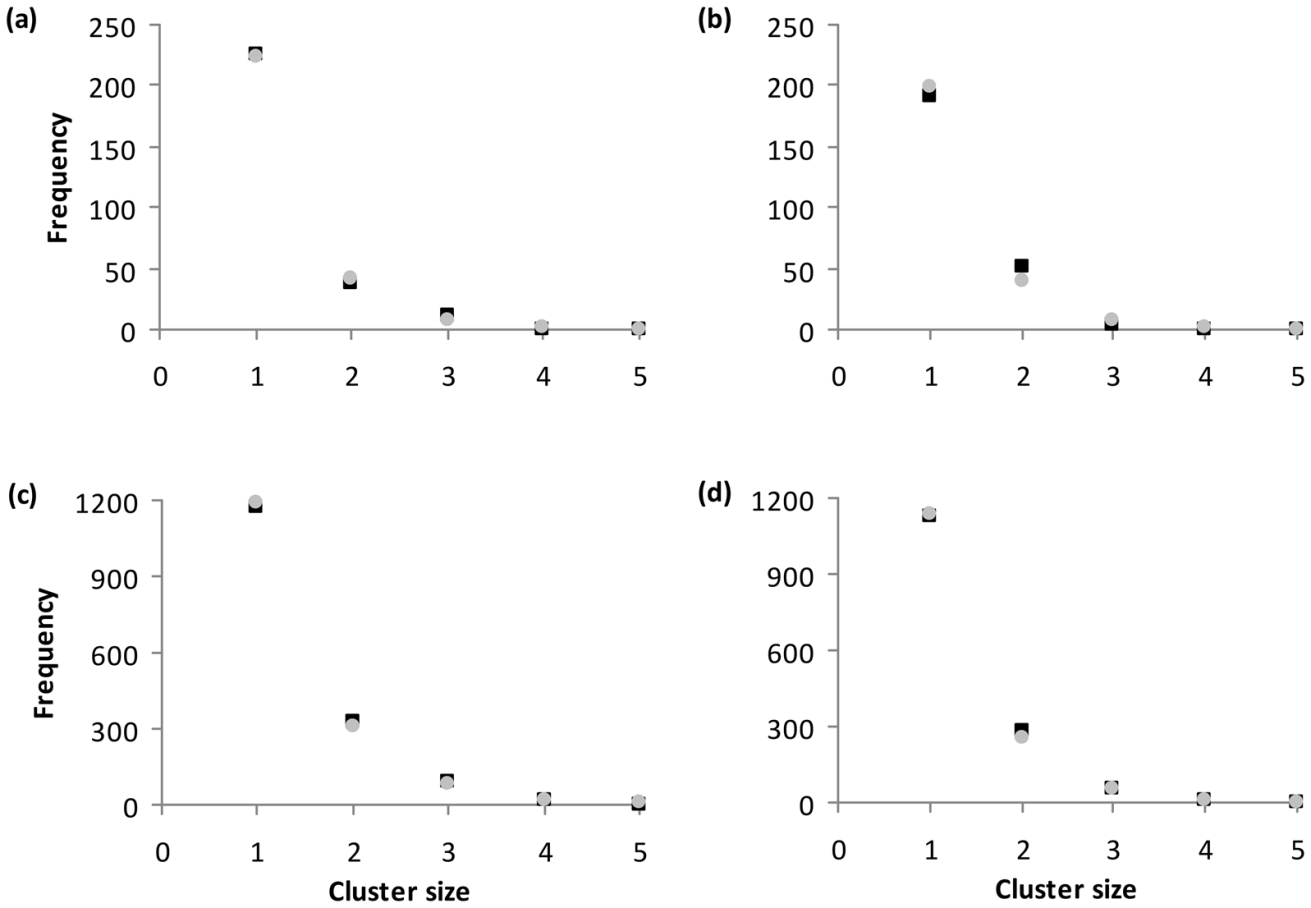
Cluster size distribution of animal sightings. Observed (black squares) versus expected (grey filled circles) cluster sizes, i.e. the numbers of different species in the clusters, for (a) TME wet, (b) TME dry, (c) SNE wet, and (d) SNE dry. The observed mean cluster size was 1.223 (1.243) in TME during wet (dry) season, and 1.346 (1.291) in SNE during wet (dry) season.

Our simulation takes into account the relative frequencies of occurrences of the species, the distribution of cluster sizes (i.e. of the numbers of species per cluster), and the association strengths of pairs of species. We estimated the parameters that characterise these three factors from our observations and used 4 parameter sets, one for each pair of ecosystem and season. The observed frequencies of cluster sizes, i.e. the numbers of different species in the clusters, can be described by a geometric distribution for both ecosystems and seasons (Figure 2). Only species with a high detectability were taken into account (9 species in TME and 10 species in SNE). We used a geometric distribution where the probability of cluster size *n* followed the formula *P*(*n*)  =  (1-*p*)*^n^* ^-1^ *p*. We estimated the parameter *p* for each ecosystem and season by taking the inverse of the respective sample mean of cluster sizes. The simulation consists of three steps. In step 1, *N* occurrences (i.e. sightings) of species are generated by drawing species with replacement using probabilities according to the relative frequencies of the species. In step 2 clusters are formed by repeatedly generating a new cluster and filling it with species occurrences drawn without replacement from the *N* occurrences generated in step 1 (avoiding multiple occurrences of a species in the same cluster) such that the resulting cluster sizes follow the above described distribution. Step 2 ends when all species occurrences have been assigned to single or mixed species clusters. If the total number N of generated occurrences of species is set to the total number of actually observed occurrences, steps 1 and 2 generate sets of clusters which, on average, reproduce the observed absolute frequencies of the species as well as the observed numbers and sizes of the clusters. In step 3 preferences are introduced by performing *k* random swaps of pairs of species from different clusters, where a swap is immediately reversed if it increases the difference in association strength between the set of clusters and the observation. For our analysis we set *k* to 10000 (other values >1000 led to very similar results). The swapping operations retain the cluster sizes and the numbers of occurrences of the species but produce co-occurrences of species that are closer to the observed ones than those immediately after step 2. The difference in association strength was measured in the following way: for each pair of species the relative association strength in the simulated (observed) set of clusters is computed by dividing the number of co-occurrences by the total number of co-occurrences of all pairs of species in the simulated (observed) set of clusters. Then the sum of squares of differences between the relative association strengths in the simulated and in the observed set of clusters of all pairs of species is computed.

If step 3 is omitted, the simulation generates preference-free clusters which can be used to investigate the influence of the existing preferences of the species on the network measures. We can then see whether the betweenness of a species is solely explained by the species' abundance, or whether the species' preferences also play an important role. If step 3 is included, the simulation can be used to generate clusters that follow the same patterns as our observations. In order to derive meaningful comparisons between ecosystems and seasons, we evaluated ranks of the scores rather than raw values. By repeating the simulation multiple times (N = 1000) we constructed a number of potential networks that constitute likely variants of the observed network. Therefore, the spread of the (ranks of the) values of the network measures indicates the robustness of conclusions regarding specific network positions.

## Results

### Species-specific association patterns

**Figure 3:**
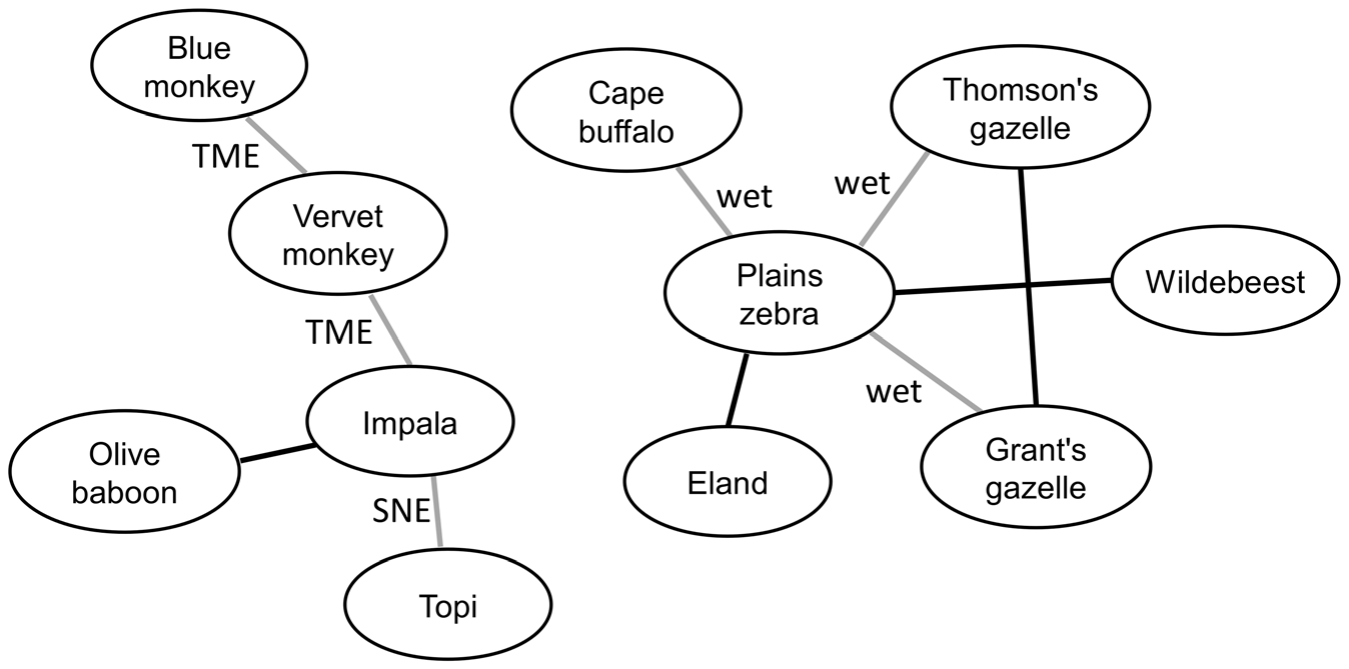
Significant associations among large mammal species in the Tarangire-Manyara (TME) and the Serengeti-Ngorongoro ecosystems (SNE). Black edges denote significant associations in both ecosystems and both seasons. Grey edges illustrate species associations that were significant in one ecosystem (“TME”, “SNE”) or in one season (“wet”).

Considering all species, we found general non-random associations in the data for each ecosystem and season (BC test, N = 10^7^ randomization steps, test statistic  =  ASSS score, all 4 p<0.001). Considering specific pairs of species (Fig. 3) we found that the following pairs were associated in each ecosystem and season: plains zebra - eland, plains zebra - wildebeest, and impala - olive baboon (BC test, N = 10^9^ randomization steps, test statistic  =  number of co-occurrences, p: 0.0001–0.012). This also holds for the pair Grant's gazelle - Thomson's gazelle, except for TME during dry season. Consistent association patterns across ecosystems or seasons were also found for plains zebra – cape buffalo, plains zebra - Grant's gazelle, plains zebra - Thomson's gazelle (during wet seasons in TME and SNE, p: 0.0001–0.029), impala - vervet monkey and vervet monkey - blue monkey (TME during wet and dry season, p: 0.0004–0.021), and impala - topi (SNE during wet and dry season, p: 0.001–0.005). When additionally controlling for habitat types, randomisation results showed the same patterns.

Kirk's dik-dik, African bush elephant and Maasai giraffe had small node strength in each ecosystem and season (BC test, N = 10^8^ randomization steps, test statistic  =  node strength, all p<0.025), suggesting they occurred less often in mixed species groups than expected given their sighting frequencies. Wildebeest and plains zebra had high node strength in each ecosystem and season (all p<0.025, but p = 0.049 for plains zebra in TME during the wet season). This shows that both species not only have preferences that lead to associations with certain other species but also tend to generally occur in mixed species groups. In SNE, many carnivores (serval, leopard, cheetah, lion) tended to occur in single species clusters (all p<0.048, except for serval during the dry season p = 0.109). During the wet season spotted hyenas occurred frequently in mixed species groups (p = 0.042), particularly with Cape buffalo, hippopotamus, wildebeest and plains zebra. In TME carnivore sightings were infrequently observed, leading to low test power.

### Group size patterns

Impala, wildebeest (SNE wet season), and Defassa waterbuck (SNE dry season) had smaller group sizes when they occurred in single- versus multi-species clusters (N = 10^5^ permutations, p<0.036 in a two-sided test). Kirk's dik dik had larger group sizes in multi-species clusters in TME during the wet season (N = 10^5^ permutations, p = 0.047 in a two-sided test). In other species group sizes did not differ between cluster types. For most pairs of significantly associated species the group sizes did not differ whether the species co-occurred or not. However, both wildebeest and plains zebra had larger group sizes when they co-occurred in SNE (wet and dry season, N = 10^5^ permutations, p<0.009 for both species) and in TME (dry season, N = 10^5^ permutations, p = 0.004 for plains zebra, and p = 0.056 for wildebeest). For other species combinations, group size differences were rare (5 significant signals in 72 cases) and never consistent across ecosystems or seasons. This suggests that species did not necessarily trade-off conspecifics for heterospecifics, but that heterospecific associates were added over-and-above the local conspecific associates, resulting in overall larger groups.

### Betweenness of mammal species

**Figure 4:**
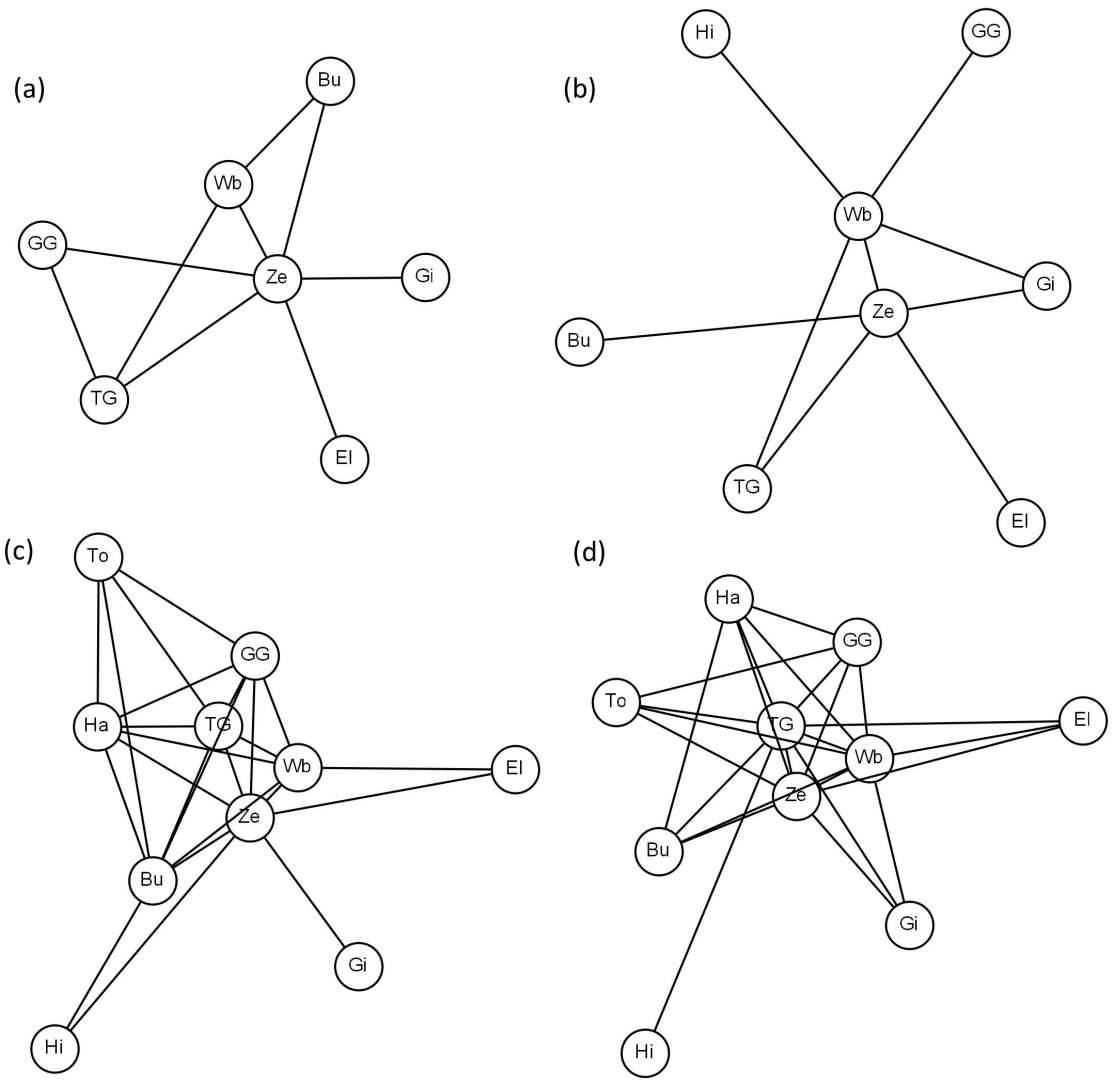
Observed networks of large mammals. Networks were restricted to species (see Table 1 for abbreviations) with high detectability and a minimum of two co-occurrences for (a) TME wet, (b) TME dry, (c) SNE wet, (d) SNE dry. The layout of the networks follows a spring model, where the lengths of the edges correspond to the association strengths. A short edge indicates a high numbers of co-occurrences. The sampling effort differed between TME and SNE; therefore the networks are not directly comparable between the ecosystems. The networks were drawn using Graphviz version 2.38.

**Figure 5:**
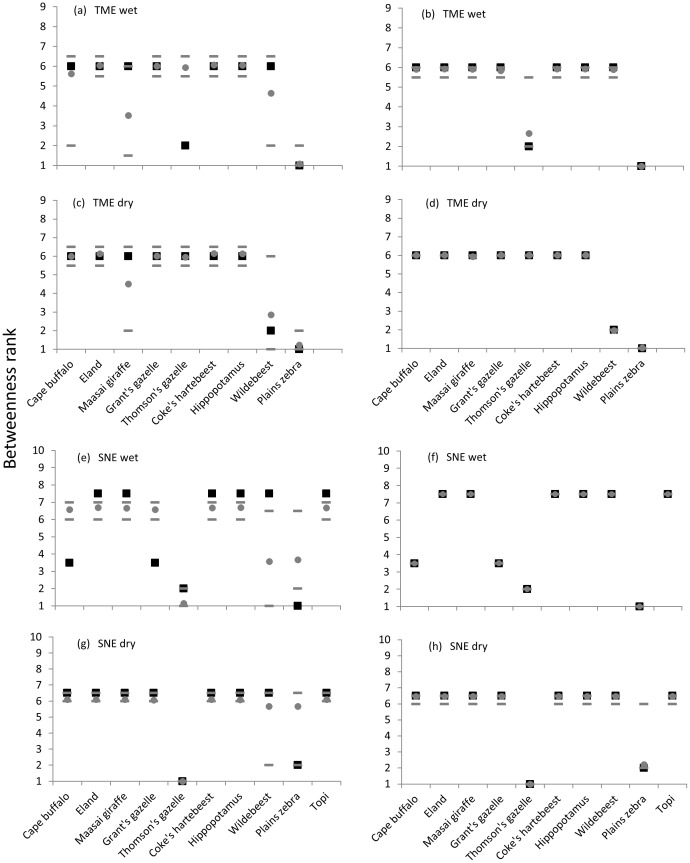
Betwenness ranks of species derived from simulated networks. Observed (black squares) versus expected betweenness score ranks (grey filled circles, lines depict the 5% and the 95% percentiles) when networks were simulated based on relative abundance of species only (left panel) and when taking heterospecific preferences into account (right panel).

Figure 4 illustrates networks of species with high detectability for both ecosystems and seasons. Visually, each of these networks indicates a central role of zebras and indeed zebras always scored high betweenness ranks (Fig. 5). In order to further investigate the species-specific betweenness, we conducted simulations as described in the methods. When the simulation included the preferences, variations in the numbers and sizes of clusters, frequency of occurrence of the species, and number of co-occurrences lead to very small variations in the betweenness ranks (Figs 5b, 5d, 5f, and 5h), suggesting that our observations provided a sufficient basis for the construction of robust networks. The simulations also show that both the abundances and the preferences of species influence their betweenness (Fig. 5). Plains zebra had a consistently high betweenness rank across both ecosystems and seasons (Figs 5b, 5d, 5f, and 5h). In TME this can be solely explained by the high abundance of this species (Figs 5a, 5c), while in SNE the plains zebra's preferences were important as well (Figs 5e, 5g). In contrast, the wildebeest, which also had a high relative abundance, only had a high betweenness rank in TME during the dry season. This is particularly interesting in SNE, where its expected mean rank is similar to that of the plains zebra if only abundances are taken into account. The Thomson's gazelle had a high betweenness rank in SNE during both seasons due to its high abundance. It also had a high betweenness rank in TME during the wet season in spite of its relatively low abundance, which can only be explained by its preferences. Similarly, Cape buffalo and Grant's gazelle had betweenness ranks higher than could be explained by their medium abundance alone in SNE during the wet season. In contrast, Maasai giraffe in TME, in particular during the wet season, had a lower betweenness rank than suggested by its relatively high abundance.

## Discussion

In the two studied savannah ecosystems, most mammal species tended to form mixed species groups. Notable exceptions were elephants, giraffes, dik-diks, and most carnivores; these species seemed to avoid proximity to other species. Several dyadic associations appeared to be consistent across ecosystem and season (baboon-impala, eland-zebra, Grant's gazelle-Thomson's gazelle, wildebeest-zebra) while other associations were either only found in one ecosystem (TME: blue monkey-vervet monkey, vervet monkey-impala; SNE: impala-topi) or during the wet season only (Cape buffalo-zebra, Thomson's gazelle-zebra, Grant's gazelle-zebra). During the dry season, the betweenness ranks in mammal networks were sufficiently explained by the relative abundance of mammal groups whereas during the wet season, social preferences of the species were important for the observed mammal networks. These findings may suggest that mixed species aggregations are a consequence of scarce and highly heterogeneous resource distribution in the landscape in the dry season [65], [66]. Yet, during the more resource abundant wet season, heterospecific attraction or avoidance apparently affected the formation of animal networks in addition to the relative abundance of the different species. This underlines a previous hypothesis that mixed species groups emergence is an outcome of interspecific resource competition during the dry season and driven by anti-predator advantages during the wet season [67].

In fact, some species appeared to be more central within the network than expected during the wet season (TME: Thomson's gazelle; SNE: Cape buffalo, Grant's gazelle, zebra) (Fig. 5). This seasonal shift in association patterns may be partly an outcome of seasonal shifts in grazer-specific food preferences [68] which leads to the partitioning of grass species and grass heights and thus governs the spatio-temporal distribution of grazers and thus co-occurrences of different mammal species [69]-[72].

Beyond resource distribution, multiple other hypotheses have been suggested to explain association patterns of mammals. For example, mixed groups may form because of similar step lengths [73] and body size may also be associated with the formation of mixed species groups [74]. Interestingly, two of the largest species (elephant and giraffe) and the smallest antelope (dik-dik) were rarely observed in mixed species groups. Yet, as body size is closely associated with the social system and the feeding strategy of East African mammals [69], [75], it remains difficult to attribute mixing patterns to body size *per se*. It has often been postulated that mixed groups have evolved from a trade-off of behavioural costs and benefits [69] and, interestingly, both plains zebra and wildebeest groups were frequently larger when found together, suggesting that group size trade-offs in both species are mediated by this pairwise association. Yet, as our results provide a thorough overview of association patterns in large mammals inhabiting savannah ecosystems, it is beyond the scope to put specific hypothesis forward why certain species form mixed groups with other species. However, our study may provide a useful basis for studying cost-benefit trade-offs in species-specific assemblages during different seasons.

### Benefits of mixed species groups

Individuals in mixed groups may have higher survival rates because members of mixed groups may be more effective in confusing or deterring predators or competitors than individuals [9], may have augmented abilities to detect predators [38] and may dilute individual mortality risks [11]. This might be particularly important during the wet season, when the grass is high and predators may be more difficult to detect [67]. In fact, impalas have been observed to actively seek the proximity of baboons when faced with immediate predation pressure from a cheetah [76]. Several species also actively facilitate food for each other. For example, ungulates often consume food that primates drop from vegetation [31], [32]. Indeed, in TME we frequently observed impalas feeding on *Vachellia tortilis* pods as they were dropped by olive baboons. While such functional relationships obviously exist, inferring functional relationships among species from a snapshot of social association patterns remains hypothetical. Thus, descriptive network analyses need to be augmented with direct observations on the type and direction of extra-species social relationships [30], [77], [78] in particular with reference to fitness-related costs and benefits [79]. In this context, it may be interesting to study extra-species social interactions of zebras and to investigate whether zebras provide information to other species or seek information from other species [30]. In line with this idea, it would be worth investigating if individuals in mixed groups adjust their behavioural budgets and gain indirect benefits from mixed species aggregations [29]. In addition, zebras have been shown to alter their movement patterns in response to immediate predation risk imposed by lions [80]. Therefore, observing mixed grouping patterns along a predation gradient would be an interesting approach. Similarly, it may be worthwhile testing if zebra foraging behaviour is affected by ranging behaviour of associated species [77]. Such additional lines of investigations are necessary to provide a more comprehensive view on the evolution and functional significance of mixed-species groups [81].

### Costs of mixed species groups

Being in mixed groups may increase competition for food among individuals. In multi-species associations, such competition may be reduced compared to single-species groups because species may have very nuanced food preferences [68]. Yet, during times of food scarcity (i.e. during the dry season), grazing ungulates may strongly compete for the scarce remaining grass patches while during the wet season, competition may be negligible because species may select different grass species and heights [36], [37], [70]. However, as we rarely observed decreases in conspecific group sizes, competition for food may not necessarily be driving mixed-species associations and species may rather benefit from group-size augmentation. Similarly, the effect of mixed grouping patterns on predation risk may be situational. Whereas impala-baboon associations may reduce the predation risk for impalas in specific circumstances [76], baboons prey on young impala [28] and thus, being associated with baboons could be very costly for impalas during the calving season.

If animals aggregate with other species – either because of resource limitation or because of heterospecific attractions - the likelihood for spill-over pathogen transmission is clearly affected [12], [13], [50]. This is particularly true for pathogens such as the causative organisms of anthrax, bovine tuberculosis, and rinderpest that are transmitted directly from individual to individual or from animal matter to individuals [82]. The spread of directly transmitted pathogens is strongly influenced by the contact rates between infected and susceptible hosts. In biological systems, contact rates are often highly heterogeneous [83]. Networks provide a way to visualise and model these specific relationships and to draw conclusions about the roles of individuals (or specific groups), modelled as nodes, in a global context [60], [84]. For example, network measures of centrality (betweenness), may indicate the influence that certain nodes have on transmission processes. Taking network structures into consideration can therefore improve the quality of predictions regarding the spread of diseases [85]. Indeed, using a combination of microbial genetics and network techniques, VanderWaal and colleagues [50] showed that Grant's gazelle and zebra appear to play a central role in the cross-species transmission dynamics of pathogens. Particularly the central role of zebras is reflected in our study, suggesting that (a) contact networks of species are similar across study sites and that (b) observed contact networks are a useful proxy for actual pathogen transmission.

### Constructing reliable networks

In practice, sampling data for constructing networks can be difficult for a number of reasons. Some individuals or species can be more difficult to observe than others, potentially introducing a bias. Also, large networks can usually only partially be observed with regard to time and space [60]. In our study we eliminated one potential bias by restricting our network to highly detectable species. Additionally, we assessed the robustness of our networks by comparing them with networks constructed from simulated ‘observations’ that followed basic patterns regarding the formation of clusters which we derived from our original observations. Understanding the processes underlying social networks is generally important for deciding whether a network sample was constructed from a sufficient amount of data or whether its structure is likely to change when more data is added. In the latter case, conclusions have to be drawn very carefully. In line with this, we consider working with weighted networks crucial when studying pathogen transmission, because they explicitly consider contact frequencies. Advanced approaches of ‘reality mining’ have the potential to address some of these issues by using automated technologies for the collection and processing of data allowing the construction of complex, realistic social networks as well as dynamic models that describe their underlying processes [86].

## Conclusion

Factors affecting mixed species grouping patterns apparently differed between dry and wet season, suggesting that mixed species groups during the dry season may be caused by decision processes that are independent of other species [87] while they are probably an outcome of specific cost-benefit trade-offs during the wet season [67]. In order to investigate the costs and benefits of species associations, tailored behavioural studies need to assess the species-specific implications of extra-species associations. Because animal network characteristics appear predominantly affected by the relative abundance of species, changes in species specific densities could largely affect species interactions. If individuals in mixed groups gain important information from other species, population declines in information providing species could have severe effects on information receiving species [30]. At least partly, the loss of such heterospecific interactions may play a role in the often observed cascading effects that follow single-species extinctions. Thus, gaining a better understanding of interactions in mixed species groups may be crucial for predicting consequences of extinctions or introductions of species in animal communities [88].
